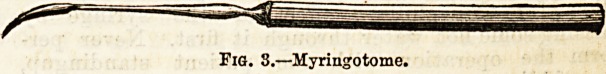# On the Treatment of Otorrhœa in the Aural Department

**Published:** 1893-10-14

**Authors:** L. H. Pegler


### The Hospital Clinic.

[*The Editor will be glad to receive offers of co-operation and contributions from members of the profession. All letters should be addressed to* The Editor, The Lodge, Porchester Square, London, W.]

### ST. MARY'S HOSPITAL, LONDON.

### On the Treatment of Otorrhœa in the Aural Department.

From the year 1851, when Toynbee was appointed aural surgeon to the above institution, up to the present date, the department devoted to the ear has been presided over by a series of distinguished surgeons, and has enjoyed a well-deserved notoriety. It will prove both interesting and instructive to inquire briefly into the methods adopted by Mr. G. P. Field and his assistant Dr. Wm. Hill at St. Mary's Hospital, in the management of uncomplicated aural suppuration. By limiting ourselves to the simple form of the disease, we shall be excluded from a consideration of the serious lesions that often attend upon or follow it; such, for instance, as aural polypus, mastoid abcess, caries and necrosis of the temporal bone and meningitis.

### I. Acute Suppuration.

In acute inflammation of the middle ear—technically known as acute *otitis media*—as in many other diseases, prevention is better than cure, and it is a not uncommon occurrence to find patients presenting themselves in so early a stage of the inflammatory mischief that by timely preventive methods the intervention of suppuration can be averted. With this object, depletion, both local and general, is the first procedure to have recourse to, and just as in ophthalmic practice leeches are applied to the temples for the relief of pain and inflammation in the eye with the utmost benefit, so in aural surgery a like result may be obtained by drawing blood from the immediate neighbourhood of the meatus. On anatomical as well as empirical grounds, it has become the rule to select the tragus as the most suitable site, and not less than from four to six leeches should be applied in succession. To confine them to the precise spot indicated is of importance, and hence, after preparing the surface in the customary way, a conveniently sized test tube will be found to be the best recipient for the leech, which can thus be prevented from wandering too far away towards the cheek. Nothing is more disappointing than to find after ordering this mode of depletion that the result has been unsatisfactory owing to neglect of this precaution.

It occasionally happens that pain and tenderness are complained of over the region of the mastoid cells, even in a very early stage, and in that case the space immediately behind the ear is chosen for letting blood. Not less than six leeches should be employed, and in this case they can conveniently be confined to the part in a chip box, if a sufficiently large one be chosen.

General depletion, especially in full-blooded or constipated habits, is to be effected by the administration of a saline purgative, the ordinary white mixture answering the purpose very well. The following is a useful adjunct for the relief of pain: ℞ acid carbolic, cocain hydrochl. aa. gr. v., glycerini ℥j. Sig. To be instilled warm into the ear as often as necessary, from five to forty drops or more being used at a time.

In the intervals great relief is often obtained by gently syringing or pouring into the ear water of a temperature as hot as can be borne with comfort; but hot poultices of whatever kind are best avoided. When, in spite of these measures, the drum-head is seen to be red and bulging, or in the absence of this indication, when pain obstinately persists, it is safer and wiser to puncture it by making a free incision in its posterior segment, than to leave it to rupture spontaneously. (The drawing of the appearance presented by the normal drum head indicates the various structures seen and referred to in the text.) The result of the incision is more or less free bleeding, but although pus does not always make its appearance at first, a serous fluid follows, which in a few hours becomes purulent; so that absence of pus in the first instance is not to be taken as an indication that the operation has been performed prematurely. As cocaine is unfortunately quite ineffectual in anæsthetising the surface of the membrana tympani, it devolves upon the attendant to render every assistance in steadying the head and hands of the patient when the incision is being made, as the latter is almost sure, on the first contact of the paracentesis knife, to endeavour to arrest the surgeon's hand, or jerk his head out of reach, thus rendering the operation

Fig. 1.

Fig. 2.

Fig. 1.—The appearance presented by the normal right membrana tympani. 1, The membrane; 2, the handle of the malleus; 3, short process; 4, posterior, and 5, anterior folds; 6, membrana flaccida; 7, bright spot.

Fig. 2.—The appearance presented by a depressed membrana tympani on the right side. The handle of the malleus is foreshortened and the short process very prominent. The posterior fold is very marked.

both dangerous and ineffectual. For this reason in nervous persons it is preferable to give a general anæsthetic. The strictest antiseptic precautions should be followed in the operation of puncturing the drum head, the external meatus being first syringed out with a warm 1 to 20 solution of carbolic acid, and the knife scrupulously disinfected in the same solution, or by immersing the blade in absolute alcohol. When the incision has been made, the contents of the tympanic cavity are to be evacuated by the use of Politzer's air-bag, after which a plug of boric wool may be inserted into the meatus, and renewed frequently so long as bleeding continues.

Fig. 3.—Myringotome.

Before dismissing this subject it will be convenient to state the importance of examining the condition of the membrana tympani in all cases where acute pain on one side of the head or face is complained of, and this, whether febrile symptoms exist or not, as it frequently happens that no allusion is made by the patient, directing the surgeon's attention to his ear. The radiation of the pain along the sensory branches of the fifth nerve (which communicate freely with the facial) in their distribution over the cheek and temple causes the patient to describe his ailment as an attack of neuralgia or face ache. The practical import of this point becomes manifest when it is remembered that lives are sometimes endangered through neglect of the performance of paracentesis at the moment it is indicated, meningitis supervening upon the tension created by the confinement of effusion in the tympanic cavity with fatal consequences.

Supposing a case presents itself in which rupture of the membrana has recently occurred spontaneously, it will be found on inquiry that the discharge has been purulent from the commencement, and its glairy character and complete freedom from fœtor will always indicate its recent history. On syringing this away the membrana tympani will be seen; it is difficult to cleanse, a quantity of white sodden epithelial *débris* adhering to its surface, which when exposed is of a dark—almost bluish—red colour, tumified and glistening. The object in view now is a perfectly free drainage for the pus whilst it is profuse; the arrestment of discharge at this stage is contra indicated, yet in favourable cases, by observance of the following plan of treatment, the perforation is almost sure to close without a perceptible cicatrix, or diminution of hearing power. If the temperature is raised the patient is best confined to bed, or to the house, till it subsides. If pain continue leeches must be again applied, and now preferably to the mastoid process, and they may be repeated at intervals until it abates. Fortunately this is not often necessary, and our main reliance is placed upon frequent syringing with warm water containing a little boric acid in solution, with the use of Politzer's bag, once a day, to blow out accumulations of pus through the perforation. A small plug of absorbent wool should be placed constantly in the meatus, and frequently changed; by this means we get a very good idea of the amount of the discharge, and at the same time prevent its running over the patient's face, or on to the pillow when he is lying in bed. We also, thereby, hinder the entrance of septic organisms into the tympanic cavity. There is no necessity to smother the head and side of the face in flannels or bandages. A word here as to the best method of syringing the ear, as this operation has been already more than once alluded to. For the removal of purulent secretions an ordinary glass syringe answers sufficiently well; select one with an uniform calibre throughout, if possible. Glass syringes, besides being cheap and cleanly, have an advantage over the india rubber ball-syringes in many respects. They can be more accurately pointed in the direction required, and all air can be excluded from them when charged by holding them vertically and pushing up the piston till nothing but the solution remains. This saves the patient some annoyance. It will be understood that we are here referring to syringes for home use. In hospital the brass syringe with movable nozzle is invariably employed; but whichever kind we use, it is well in cases of acute sappuration, to attach to the nozzle an inch or so of narrow indiarubber tubing; this enables one to penetrate further into the meatus, and therefore wash out more thoroughly, without inflicting pain on the tender and inflamed parts. Let the solution used be sufficiently warmed, and allow for the chilling effect of the syringe by passing some hot water through it first. Never perform the operation with the patient standingup, as giddiness and faintness often result, and take care to allow as much light as possible to fall upon the entrance to the auditory meatus. Bear in mind the direction the latter takes, forwards, in a line with the petrous portion of the temporal bone, or, in other words, towards the pharynx, and holding the auricle back with the index finger and thumb of the left hand, direct the nozzle of the syringe forwards and slightly upwards, in order to surmount the convexity that occurs in the floor of the meatus about half an inch from the orifice. In this way a stream is directed towards the roof at the extreme end of the meatus, and foreign substances of whatever kind, whether introduced from without, or secreted from within, are pushed backwards towards the operator and extruded. Every one of these simple injunctions is important, for frequently the surgeon finds that his recommendations with respect to cleansing the ear, whether in the wards or in the patient's home, have not been intelligently carried out.

After a few days of this treatment a marked alteration will be observed in the amount of the discharge; whilst as regards hearing power, the watch, which at first could probably be only heard on contact, will be distinguished on the affected side at a few inches distant. A useful agent to employ now is the following: ℞ acid boric. gr. x.—xxx.; aq. distill., ℥j. Sig.: a few drops to be warmed by immersing the containing bottle in hot water, and poured into the ear after syringing. But if pain be complained of, lead and opium is substituted: ℞. Tr. opii, ʒss—ʒj.; plumbi acetat, gr. j.; glycerini, ʒj.; aquæ ad. ℥j. Sig.: to be used like the last. The astringency, combined with the soothing effect of this lotion, render it highly serviceable, and it is always much appreciated by patients. In the management of acute as well as of chronic otorrhœa, a watchful eye should always be kept by those in attendance against the supervention of cerebral manifestations. The presence of headache, drowsiness, vomiting, rigors or feverishness, redness or tenderness on pressure over the mastoid process, should be carefully noted, and the facts communicated to the surgeon. Free drainage we must strive for, tension we must abhor, and here let us remark upon the great variation met with in regard to the length and calibre of the external auditory canal. In some people it is extremely narrow, in others it presents in addition a strong curve in an upward direction; now, tumefaction of the meatal walls, roof, and floor is a very common accompaniment of acute suppuration of the middle ear, and when this occurs in a case in which the above conformation is the normal, the lumen of the tube is so much contracted that nothing much thicker than an ordinary probe can be made to pass, and free vent for morbid secretions is proportionately interrupted. It is under these circumstances that invasion of the mastoid cells and cerebral meninges by the inflammatory process is especially apt to take place, giving rise to the symptoms just enumerated, and necessitating one or other of the recognized operations for their relief. The Eustachian channel probably renders some service as a drain when the secretions are fluid, but the fact of its commencing on a level slightly above the floor of the tympanum, and possessing an hour-glass constriction at about the middle of its course,can scarcely do otherwise than prejudice its usefulness in this regard. The consideration ofchronic otorrhœa will be dealt with in a subsequent article.

L. H. Pegler, M.D.

## Figures and Tables

**Fig. 1. Fig. 2. f1:**
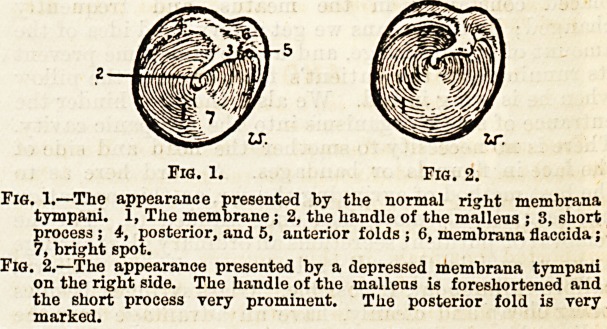


**Fig. 3. f2:**